# IL-36 cytokines imprint a colitogenic phenotype on CD4^+^ T helper cells

**DOI:** 10.1038/s41385-022-00488-w

**Published:** 2022-02-17

**Authors:** Gemma Leon, Yasmina E. Hernandez Santana, Naoise Irwin, Eirini Giannoudaki, Sadhbh O’Neill, Ilona Csizmadia, Martina Gogarty, Tae J. Lee, Darren Ruane, Aideen Long, Padraic G. Fallon, Seamus Hussey, Patrick T. Walsh

**Affiliations:** 1 Trinity Translational Medicine Institute, School of Medicine, Trinity College Dublin, Dublin, Ireland; 2 National Children’s Research Centre, CHI Crumlin, Dublin, 12 Ireland; 3 Janssen Research and Development, Spring House, PA USA; 4 Trinity Biomedical Sciences Institute, Trinity College Dublin, Dublin, Ireland

## Abstract

IL-36 cytokines are emerging as potent orchestrators of intestinal inflammation and are being implicated in the pathogenesis of inflammatory bowel diseases (IBD). However, the mechanisms through which these cytokines mediate these effects remain to be fully uncovered. Here, we report specifically elevated expression of IL-36α, and not IL-36β or IL-36γ in the serum of newly diagnosed, treatment naïve, paediatric IBD patients and identify T cells as primary cellular mediators of IL-36 responses in the inflamed gut. IL-36R expression on CD4^+^ T cells was found to promote intestinal pathology in a murine model of colitis. Consistent with these effects, IL-36R can act as a potent instructor of CD4^+^ T cell differentiation in vivo, enhancing Th1 responses, while inhibiting the generation of Tregs. In addition, loss of IL-36 responsiveness significantly reduced the migration of pathogenic CD4^+^ T cells towards intestinal tissues and IL-36 was found to act, uniquely among IL-1 family members, to induce the expression of gut homing receptors in proinflammatory murine and human CD4^+^ T cells. These data reveal an important role for IL-36 cytokines in driving the colitogenic potential of CD4^+^ T cells and identify a new mechanism through which they may contribute to disease pathogenesis.

## Introduction

Inflammatory Bowel Disease (IBD) is a chronic inflammatory disorder of the gastrointestinal (GI) tract. It is typically subcategorised into two physiologically distinct disorders, Crohn’s Disease (CD), in which pathology can present anywhere along the GI tract, and Ulcerative Colitis (UC), a condition which mainly presents in the colon.^1^ In western society incidence of this disease is on the rise, and ~25% of cases arise in childhood or adolescence.^1–3^

In both adults and paediatric patients, the ultimate goal of current treatment strategies is to offer symptomatic relief and induce and maintain remission. While historically approaches to achieve these endpoints have involved the use of broad spectrum immunomodulators, such as steroids and mesalamine compounds, an increase in understanding of the inflammatory mechanisms associated with disease pathogenesis has underscored the development of more specific biotherapeutic approaches.^4,5^ Central to these advances has been a deeper understanding of the roles of cytokines in driving intestinal inflammation, leading to the development of strategies aimed at inhibiting TNF and IL-12/23 which have benefited millions of patients.^6,7^ Despite such progress, a significant number of patients remain unresponsive to these interventions and loss of initial patient responsiveness is also common. As a result, new approaches and targets are required to meet what remains a large burden of unmet need.

The IL-36 family of cytokines is a relatively new member of the IL-1 superfamily, and since their discovery two decades ago, they have gained significant attention for their involvement in numerous inflammatory and autoimmune conditions. The family is comprised of three agonistic ligands, IL-36α, IL-36β and IL-36γ, and one receptor antagonist, IL-36Ra, which all signal through the IL-36R.^8,9^ Similar to other IL-1 members, IL-36 cytokines are generated in a biologically inactive “pro” form, that require proteolytic processing, specifically truncation of their N-termini by neutrophil granule derived proteases, to enable full activity.^10,11^ Dysregulation of IL-36 members has been associated in the pathogenesis of various conditions, such as psoriasis,^12^ rheumatoid arthritis,^13^ SLE^14^ and importantly, IBD.^15^ Recent work from our own group, and several others, have identified IL-36 family cytokines as important mediators of intestinal inflammation.^15–21^ During the earliest phases of disease-onset, we demonstrated significantly elevated expression of IL-36α in the colonic mucosa of paediatric UC patients, while deficiency in IL-36R signalling was protective against the development of colitis in preclinical studies.^15^ In the intestines, IL-36 cytokines are expressed by multiple cell types, including the intestinal epithelium and resident immune cells,^22^ and once expressed, they have the capacity to activate both innate and adaptive immune cells exacerbating inflammation.^15,18^ In contrast, IL-36 cytokines have also been reported to promote mucosal resolution and healing in the intestine.^16,19,20^ This dichotomous role for IL-36 is likely attributable to several factors, including timing, extent of tissue damage and chronicity and the preclinical models of disease under investigation. While much remains to be uncovered about how IL-36 mediates such diverse effects, we and others have demonstrated that IL-36 can influence CD4^+^ T cell responses, which contribute to the development of colitis.^15,18^ Infiltration of pathogenic CD4^+^ T cells to the intestinal mucosa is a characteristic of IBD, and accounts for much of the pathology associated with disease, rendering CD4^+^ T cells a key target in the development of novel biotherapeutics.^23–25^

In this study, we report a mechanism whereby IL-36α promotes the development of colitis through driving the generation of colitogenic CD4^+^ T cells. IL-36α expression was found to be significantly elevated in the serum of a paediatric IBD cohort and colonic T cells were identified as a potential target of IL-36 responses in these patients. Interestingly, in IBD patients, elevated serum IL-36α negatively correlated with levels of circulating IL-36Ra, indicating an environment permissive to enhanced IL-36R activation during the earliest stages of disease. IL-36R signalling exhibited potent effects on Th cell differentiation, enhancing Th1 cell responses, whilst inhibiting the generation of induced regulatory T cells (iTregs) in vivo. In addition, *Il36r* expression was required to promote the capacity of pathogenic Th cells to infiltrate the colon and mediate disease and upregulated surface expression of the ‘gut homing’ integrin, α4β7. In vitro, IL-36α stimulation promoted the generation of colitogenic CD4^+^ T cells in mice and humans, with enhanced expression of α4β7, and the chemokine receptor, CCR9. Furthermore, the capacity of IL-36α to promote a colitogenic Th cell response occurred even in the presence of the tolerogenic influence of retinoic acid (RA). Collectively, these data indicate that IL-36R signalling regulates the pathogenesis of early onset IBD by directing the generation of colitogenic CD4^+^ T cell responses.

## Results

### Serum protein expression of IL-36α is enhanced and colonic T cells express IL-36R in treatment naive paediatric IBD patients

Previous findings from our group reported elevated gene and protein expression levels of IL-36α in the rectal mucosa of treatment naïve paediatric UC patients compared to healthy controls.^15^ To extend these observations, we next investigated whether protein expression of the IL-36 family members are also altered in the periphery at the earliest stages in the pathogenesis of IBD.

**Fig. 1:**
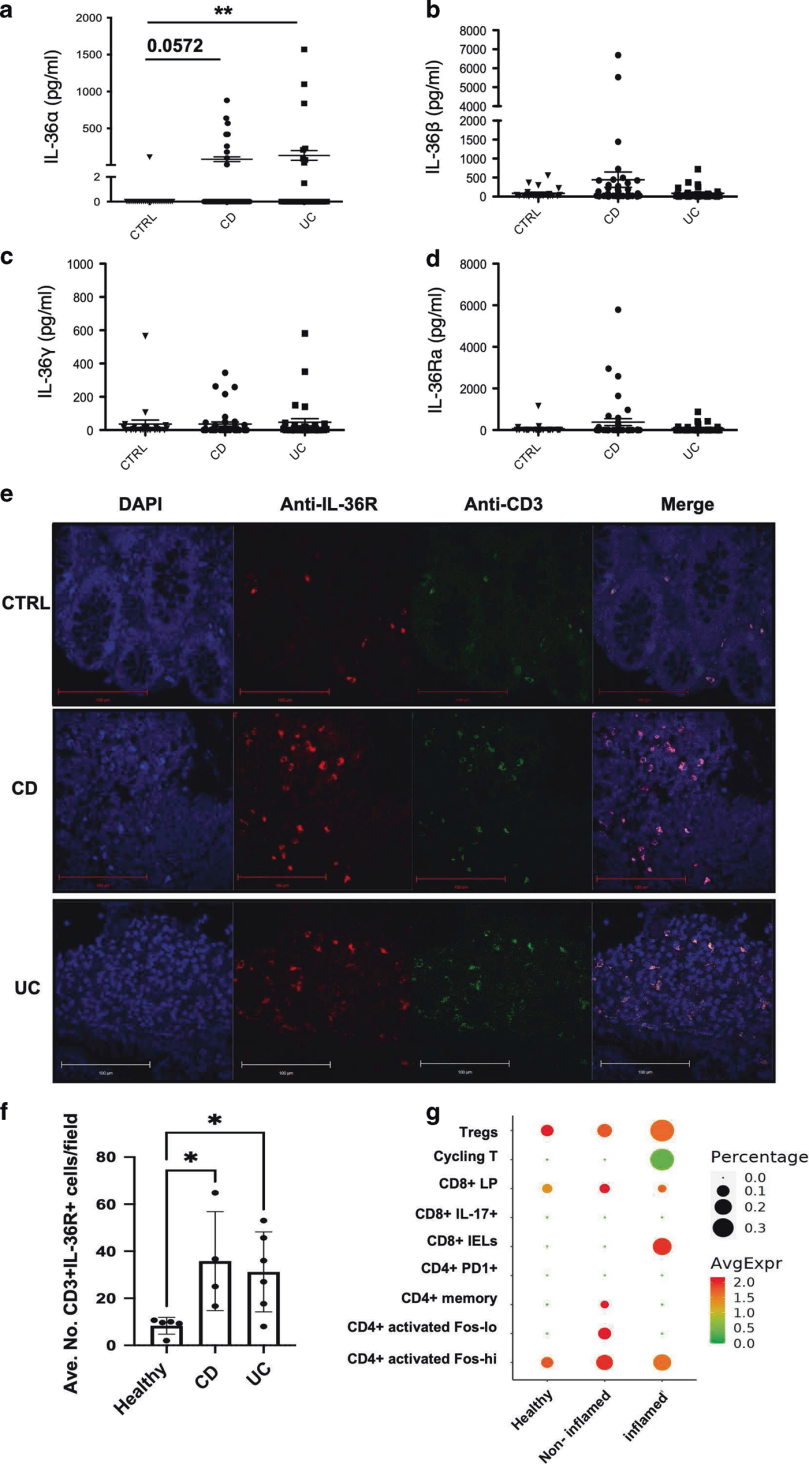
IL-36 family member expression is elevated in paediatric IBD samples. Serum samples from treatment naïve paediatric patients with CD or UC, and healthy controls, were analysed by ELISA for protein expression of IL-36α (**a**), IL-36β (**b**), IL-36γ (**c**) and IL-36Ra (**d**), *n* = CD: 41–42; UC: 31; CTRL: 23. Statistical analysis performed by Mann–Whitney U Test, **p < 0.01. **e** Detection of IL-36R and CD3 protein levels in colon biopsies from this same cohort was performed by confocal microscopy and (**f**) the average of the number of cells expressing IL-36R + and/or CD3 + per field was quantified using ImageJ (*n* = CD: 4; UC: 6; Healthy: 5). Statistical analysis performed by Kruskal–Wallis H test, *p < 0.05. **g** Analysis of scRNAseq data showing relative expression levels (heat map), and percentage frequency (circle size), of *IL36R* gene on indicated colonic T cell subsets in biopsies from healthy control (*n* = 12) and matched non inflamed versus inflamed (*n* = 18) biopsies from UC patients.

To address this, we analysed the quantity of IL-36α, IL-36β, IL-36γ and IL-36Ra proteins in the serum of paediatric IBD patients by ELISA. This analysis demonstrated a significant increase of IL-36α protein expression in the serum of UC patients compared to controls (**p < 0.01). Expression of IL-36α was also found to be elevated (*p* = 0.0572) among a subset of CD patients (Fig. 1a). While expression of IL-36β, IL-36γ and IL-36Ra was detected in the serum of all groups, no significant differences in expression levels were observed, although levels of IL-36γ, in particular, appeared to be trending higher among IBD patients (Fig. 1b–d). Importantly, these data are consistent with, and extend, our earlier gene expression-based data,^15^ demonstrating IL-36α protein expression is also increased in the serum of treatment naive paediatric UC patients, upon initial diagnosis.

Elevated IL-36α expression levels at both the site of inflammation and the periphery suggest increased responsiveness to this cytokine in the setting of IBD. In addition, a deeper analysis revealed that IBD patient serum protein levels of IL-36α were negatively correlated with levels of the IL-36Ra further indicating an environment permissive to increased IL-36 cytokine signalling in IBD (Supplementary Fig. 1A) These data prompted us to next investigate what cell types may be responsive to IL-36 stimulation in IBD. We have previously demonstrated that IL-36R is expressed predominantly by sub-mucosal lamina propria cells in the colons of IBD patients.^15^ Furthermore, we and others have previously demonstrated that IL-36 cytokines can play an important role in modulating mucosal T cell responses in murine models of colitis.^15,18^ Therefore, we investigated the potential of T cells to respond to IL-36 cytokines in the inflamed intestinal biopsies from IBD patients. To achieve this, colonic biopsies from paediatric IBD patients were stained with anti-IL-36R and anti-CD3 immunofluorescent antibodies, and their expression was analysed using confocal microscopy. This analysis indicated that higher numbers of CD3^+^IL-36R^+^ T cells were detected in biopsies from both UC (*n* = 6) and CD (*n* = 4) patients (Fig. 1e, f). In order to confirm our findings that IL-36R is expressed by colonic T cells in IBD, we also examined a separate patient cohort by analysing single cell RNA sequencing (scRNAseq) data on colonic biopsies from 12 healthy individuals and 18 UC patients as previously reported.^26^ This analysis revealed that the number of IL-36R expressing cells is increased in distinct T cell subsets present in the inflamed UC colon, including Tregs, CD4^+^ activated Fos high cells and CD8^+^ intraepithelial lymphocytes (IELs), with increased levels of *IL36R* gene expression also evident in CD8^+^ IELs (Fig. 1g).

Collectively these data reveal that there is enhanced IL-36α protein expression in the serum of paediatric IBD patients, and that colonic T cell subsets may represent an important IL-36 responding cell type at the site of inflammation in IBD.

### Deficiency in IL-36R signalling protects against the development of T cell-mediated colitis

**Fig. 2:**
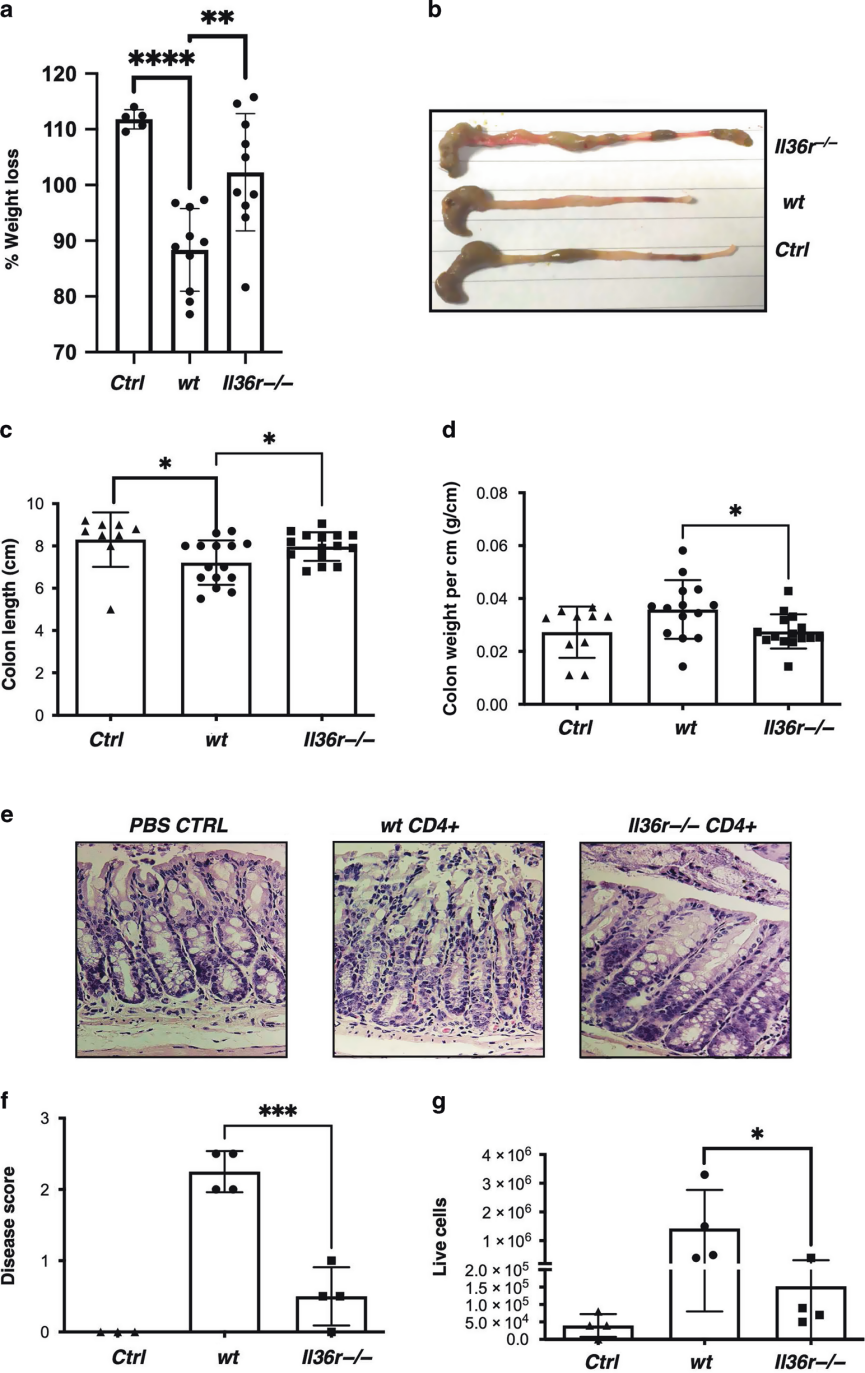
Absence of *Il36r* expression on CD4^+^ T cells protects against the development of colitis in a T cell transfer model. FACs purified CD4^+^CD25^*−*^CD45Rb^hi^ effector T cells from donor *wt* and *Il36r*^*−*^^*/−*^ mice were transferred by i.p. injection to *Rag1*^*−*^^*/−*^ recipient mice. **a** Percentage of starting weight at 4 weeks was measured to evaluate disease progression (*n* = *Ctrl*: 5; *wt*: 10; *Il36r*^−*/*−^: 10). At the experimental endpoint (week 4) colons were harvested, their length and weight per cm recorded (**b**, **c**, **d**) (*n* = *Ctrl*: 5; *wt*: 15; *Il36r*^−*/−*^: 15). Cumulative data from three independent experiments shown. H and E staining was used to determine histological disease severity (scale bar 50 μm) (**e**, **f**), and the total number of live lamina propria cells per cm of tissue was measured by Trypan blue exclusion (**g**) (*n* = *Ctrl*: 4; *wt*: 3; *Il36r*^−*/*−^: 3). Statistical analysis performed Mann–Whitney U Test (**a**, **c**, **d**) and *T*–test (**f**, **g**), *p < 0.05, **p < 0.01, ***p < 0.001, ****p < 0.0001.****p < 0.0001.

As we observed an increase in IL-36α protein expression in the serum of paediatric IBD patients, alongside the potential for this cytokine to be acting on CD3^+^ T cells in the mucosa, we next sought to investigate the specific effect of IL-36 on T cell responses in the pathogenesis of colitis. To address this, we used the T cell transfer model of colitis, in which *Rag1*^*−/−*^ mice undergo T cell driven colitis when CD4^+^ T effector cells are adoptively transferred from donor *wt* mice, in the absence of Tregs. In this model, transferred CD4^+^CD25^*−*^CD45Rb^hi^ effector T cells undergo both homeostatic and intestinal microbiome driven expansion that over time causes the development of severe colitis.^27^ To determine the effect of IL-36 signalling specifically on T cell driven colitis, we FACs sorted both *wt* and *Il36r*^*−/−*^ CD4^+^CD25^*−*^CD45Rb^hi^ T effector cells, transferred them to *Rag1*^*−*^^*/−*^ recipients, and compared the pathogenesis of disease between the groups. Mice receiving *Il36r*^*−/−*^ T cells were found to exhibit significantly less weight loss, colon shortening, colon weight (g/cm) when compared to their *wt* T cell recipient counterparts (Fig. 2a–d). This significantly reduced severity of disease was confirmed through histologic examination of colon tissues of recipient mice at the termination of the study, with increased mucosal damage observed in recipients of *wt* T cells when compared to *Il36r*^*−/−*^ T cell recipient mice (Fig. 2e, f). Overall cellularity of the colon tissues of *Il36r*^*−/−*^T cell recipient mice was also significantly reduced (Fig. 2g). These data demonstrate that *Il36r* expression on effector CD4^+^ T cells is required to directly promote the pathogenesis of colitis.

### IL-36 promotes enhanced Th1 and diminished iTreg responses in vivo

In agreement with previous reports,^18,28^ we found that IL-36 modulates CD4^+^ T cell polarisation, with enhanced Tbet/IFNγ expression by Th1 cells and reduced Th17 and iTreg differentiation (Supplementary Fig. 2). As the pathogenesis of disease In the T cell transfer model of colitis is largely associated with unrestricted Th1/Th17 CD4^+^ T cell responses in the absence of Tregs,^27^ we next decided to investigate what effect IL-36 signalling has on CD4^+^ T cell responses in vivo, in this setting. To address this, *Rag1*^*−/−*^ mice were once again reconstituted with *wt* and *Il36r*^*−/−*^ effector T cells for 4 weeks and an analysis of Th1 and Th17 signature cytokine expression by transferred CD4^+^ T cells in the colons and peripheral lymphoid organs (spleen and mesenteric lymph nodes) of recipient mice was carried out. As de novo generation of Tregs from transferred T effector cells has also been previously reported in this model,^29^ we also examined expression of the signature Treg transcription factor, FOXP3.

**Fig. 3:**
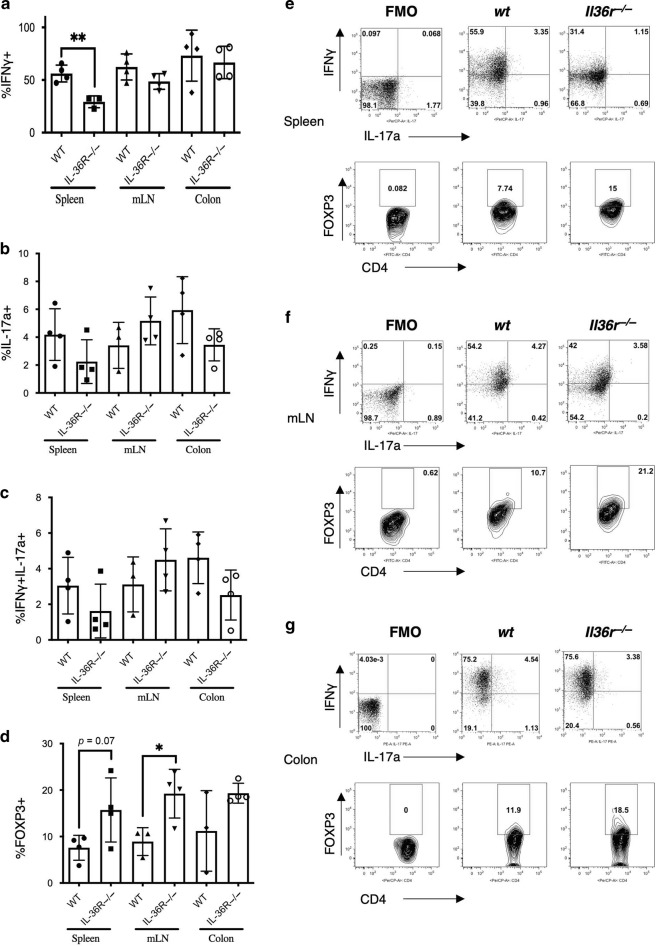
*Il36r* expression enhances pro-inflammatory CD4^+^ T cell responses in T cell mediated colitis. FACs purified CD4^+^CD25^−^CD45Rb^hi^ effector T cells from donor *wt* and *Il36r−/−* mice were transferred by i.p. injection to *Rag1*^*−*^^/*−*^ recipient mice. At week 4 the spleens, mesenteric lymph nodes (mLN) and colons were harvested for analysis of CD4^+^ T cell IFNγ (**a** and **e**–**g**), IL-17a (**b** and **e**–**g**), dual IFNγ IL-17a (**c** and **e**–**g**) and Foxp3 (**d** and **e**–**g**) protein expression by multi-parameter flow cytometry. (*n* = 3–4 mice per group as indicated). Statistical analysis performed by Students *T* Test, *p < 0.05, **p < 0.01.

The analysis of T cells revealed a significant decrease in IFNγ expression by transferred *Il36r*^−*/−*^ T cells in the spleen when compared to their *wt* counterparts, indicating a diminished Th1 response. Furthermore, a trend towards decreased IFNγ expression was observed in the mLNs of *Il36r*^−*/*−^ T cell recipient mice (*p* = 0.1), indicating that CD4^+^ T cell IL-36R signalling promotes the differentiation of Th1 responses during colitis (Fig. 3a, e, f). While there were no significant alterations in the differentiation of Th17 or dual IFNγ^+^Il17a^+^ CD4^+^responses at any site (Fig. 3b, c), a significant difference in the generation of peripherally induced Treg cells was evident. Interestingly, *Il36r*^−*/*−^ T cell recipient mice had a significantly enhanced population of FOXP3^+^ CD4^+^ T cells in both the spleen and mLNs compared to their *wt* Th cell recipient counterparts (Fig. 3d, e, f). These data are consistent with our in vitro data, demonstrating that IL-36α inhibits the polarisation of iTreg cells (Supplementary Fig. 2). While no significant differences in homeostatic baseline levels of either Th1 or Treg cells were seen under steady state conditions in *wt* and *Il36r-/-* mice (Supplementary Fig. 3), the observed alterations in Th1 and induced Treg differentiation in vivo were also evident upon co-adoptive transfer of equal numbers of *wt* (CD45.1) and *Il36r*^−*/*−^ CD4^+^ T cells (Supplementary Fig. 4A, C, D). Interestingly, the percentage of *Il36r*^*−/*−^ T cells recovered post-transfer in this setting, was significantly higher than their *wt* counterparts (Supplementary Fig. 4A–B). This indicates a cell intrinsic role of IL-36R on T effector cell differentiation under lymphopenic conditions. Taken together, these data indicate that CD4^+^ T cell specific IL-36R signalling selectively enhances pro-inflammatory Th1 differentiation while suppressing the differentiation of iTreg cells in colitogenic mice.

### Absence of IL-36R signalling results in the retention of T cells in the periphery

**Fig. 4:**
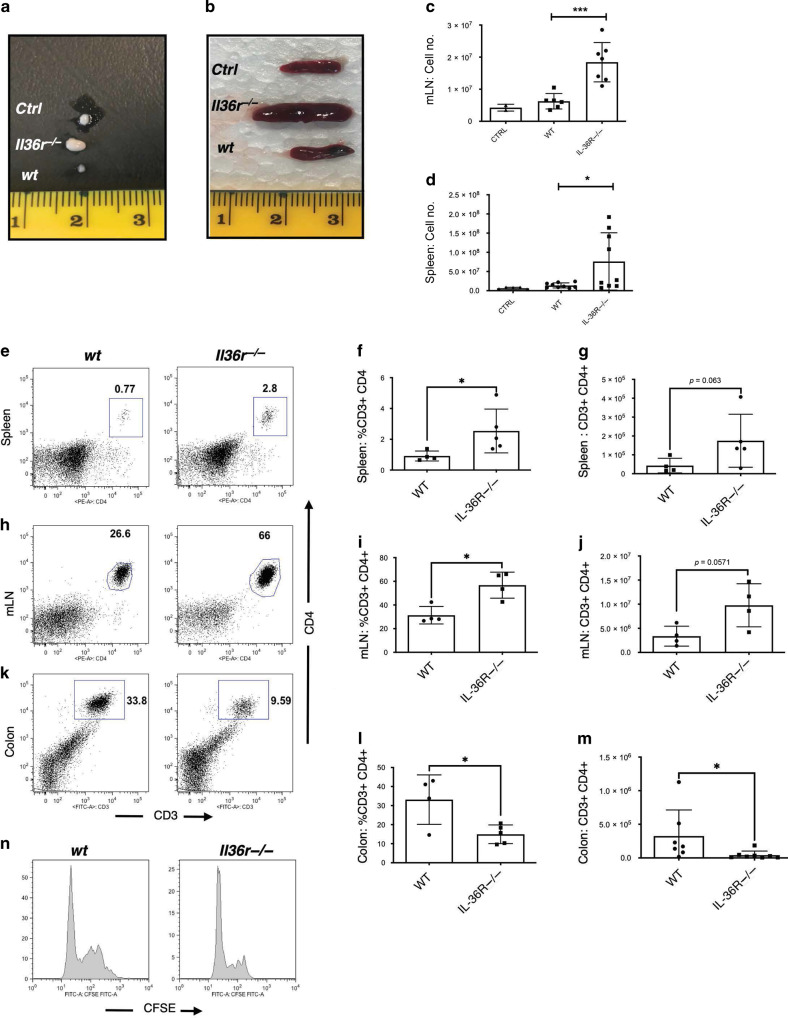
Absence of *Il36r* expression results in the development of splenomegaly and lymphadenopathy, alongside a reduced capacity for CD4^+^ T cells to migrate from the periphery to the colon. FACs purified CD4^+^CD25^−^CD45Rb^hi^ effector T cells from donor *wt* and *Il36r−/*− mice were transferred by i.p. injection to *Rag1*−/− recipient mice. PBS injected mice included as controls. Representative images of relative size of mLNs and spleens of recipient mice across all groups are shown. Scale in cm (**a**, **b**). Number of live cells present in the harvested tissues as determined by Trypan blue exclusion (**c**, **d**). Percentage and total CD3^+^CD4^+^ T cells determined by multiparameter flow cytometry in the spleen (**e**–**g**) at week 1, and the mLN (**h**–**j**) and colon (**k**–**m**) at week 4 post transfer. Data shown representative of two independent experiments with *n* = 4–8 mice per group as indicated. Relative levels of CD4^+^ T cell expansion as determined by CFSE dilution of transferred T cells after 1 week (**n**). Statistical analysis performed by Mann–Whitney U Test, *p < 0.05, ***p < 0.001.

For colitis to develop in the T cell transfer model, T effector cells must expand and differentiate into Th1/Th17 proinflammatory lineages and also traffic efficiently to the intestines, where they will initiate inflammation and mucosal destruction.^27,30^ Interestingly, whilst harvesting the tissues from recipient mice having undergone T cell transfer, we observed marked splenomegaly and lymphadenopathy in *Il36r*^−*/*−^ CD4^+^ T cell recipient mice. These mice exhibited larger mesenteric lymph nodes and spleens, when compared to their *wt* T cell recipient counterparts, and *ctrl* mice (Fig. 4a, b). Furthermore, these tissues consisted of significantly more total live cells (Fig. 4c, d), which consisted of significantly greater percentages and increased total numbers of CD3^+^CD4^+^ T cells than *wt* T cell recipient mice (Fig. 4e–j). Conversely, we observed the opposite in harvested colon tissue, where recipients of *wt* CD4^+^ T cells were found to have significantly higher percentage and numbers of infiltrating CD3^+^CD4^+^ T cells compared to *Il36r*^−*/−*^ recipient counterparts (Fig. 4k–m). One possible explanation for the observed accumulation of *Il36r*^*−/*−^ T effector cells in the periphery would be that these cells have expanded more efficiently. However, analysis of CFSE dilution of transferred cells indicated that cells proliferated to a similar extent irrespective of *Il36r* expression (Fig. 4n). These data indicate that IL-36 signalling may play a role in T cell trafficking to intestinal tissues and/or egress from the periphery. Therefore, IL-36 may have multiple roles in promoting the generation of colitis, influencing both key colitogenic mechanisms of pro-inflammatory T cell polarisation as well as migration to the intestines.

### IL-36R signalling promotes the generation of colitogenic gut homing T cells

**Fig. 5:**
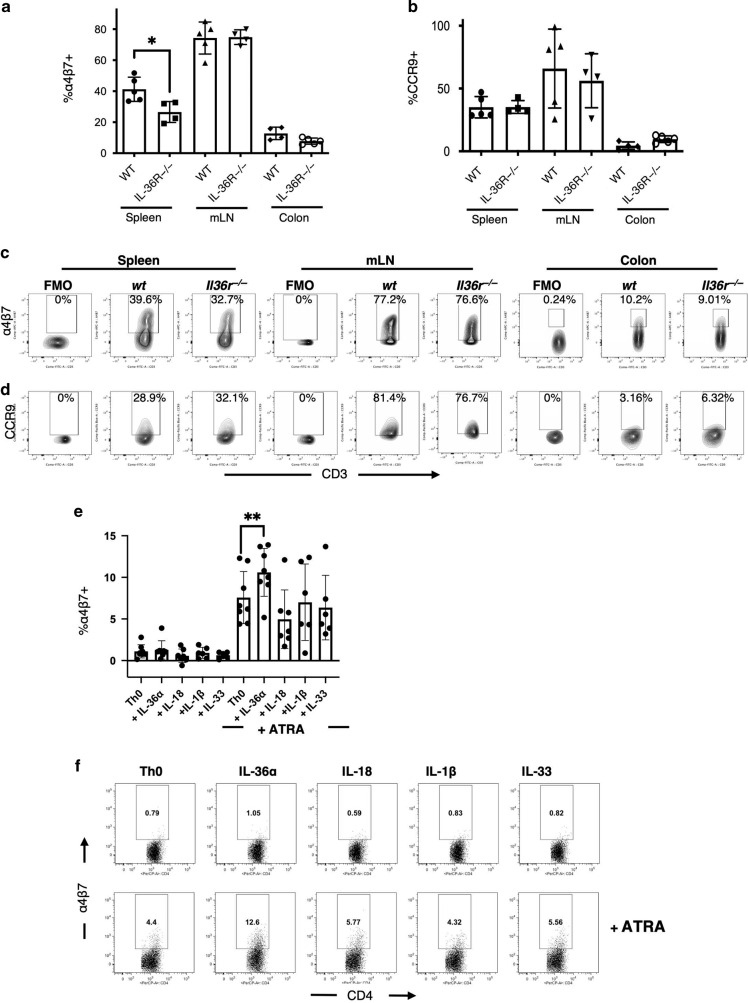
IL-36R signalling promotes the generation of murine pro-inflammatory CD4^+^ T cells with a gut homing phenotype. Flow cytometric analysis of transferred CD4^+^CD25^−^CD45Rb^hi^ effector T cells from donor *wt* and *Il36r*^−*/*−^ mice for surface expression of α4β7 (**a** and **c**) and CCR9 (**b** and **d**)(*n* = 4–5 mice per group). Statistical analysis performed by Mann–Whitney U Test, *p < 0.05. For in vitro analysis, wild type splenic CD4^+^ T cells were magnetically purified and activated with plate bound anti-CD3 and anti-CD28 and stimulated with the indicated IL-1 family cytokines; IL-36α (200 ng/ml), IL-18 (200 ng/ml), IL-1β (200 ng/ml), IL-33 (200 ng/ml); +/− ATRA (10 nM). Cells were incubated at 37 ^o^C for 72 h and analysed for expression of α4β7 (**e**,**f**) by flow cytometry. Cumulative data from two indpendent experiments is shown (*n* = 8). Statistical analysis performed by Students *T* Test, *p < 0.05, **p < 0.01.

As we had demonstrated a role for the IL-36R in regulating the infiltration of CD4^+^ T cells to intestinal tissues, we next examined whether IL-36 cytokines could regulate the gut homing capacity of CD4^+^ T cells. Previous studies have described the expression of the integrin α4β7 and the chemokine receptor CCR9 as playing important roles in mediating intestinal homing of peripheral CD4^+^ T cells.^31^ Therefore, we examined expression of both markers on transferred *wt* and *Il36r*^*−/*−^ CD4^+^ T cells in the spleens, mLNs and colons of recipient *Rag1*^*−/*−^ mice. While basal expression levels of α4β7 were similarly low on freshly isolated CD4^+^ T cells pre-transfer (data not shown), this analysis revealed a significant reduction in induced α4β7 expression by CD4^+^ T cells, in the absence of the IL-36R, in the spleens of recipient mice 1-week post transfer (Fig. 5a, c). In contrast, no significant changes in expression of α4β7 were observed in either mLN or colon tissues. Furthermore, expression of the chemokine receptor CCR9 was not found to be significantly altered on transferred *Il36r*^−*/*−^ CD4^+^ T cells when compared to *wt* cells (Fig. 5b, d). These data show that IL-36R signaling promotes the induced expression of the gut homing integrin α4β7 on CD4^+^ T cells during colitis.

Retinoic acid (RA), a vitamin A metabolite, is widely regarded as an important regulator of T cell gut homing.^32^ RA stimulation of CD4^+^ T cells is known to enhance α4β7 expression on these cells, thereby facilitating their trafficking to intestinal tissues.^32^ However, in direct contrast to IL-36, RA also inhibits proinflammatory Th1 generation, and promotes the polarisation and expansion of Treg cells,^33,34^ thereby promoting homeostasis in the gut.^33–41^ As RA is naturally present in the intestines,^42^ we next sought to compare the effects of IL-36α and RA on the induction of α4β7 on CD4^+^ T cells. To achieve this, we stimulated purified *wt* CD4^+^ T cells with IL-36α in the presence and absence of All-Trans Retinoic Acid (ATRA) under various activation/differentiation conditions.

Interestingly, this analysis revealed low expression of α4β7 on CD4^+^ T cells activated under non-polarising conditions even in the presence of IL-36α. As expected, the presence of ATRA resulted in elevated expression of α4β7, which was markedly increased further when both ATRA and IL-36α were present (Fig. 5e, f). This capacity to enhance α4β7 (and IFNγ) expression was shared by all IL-36 cytokines (IL-36α/β/γ) (Supplementary Fig. 5) but was not observed for any related IL-1 family members (IL-1, IL-33 and IL-18) (Fig. 5e, f). Therefore, IL-36α has the capacity to directly induce a gut homing phenotype on activated CD4^+^ T cells in the presence of ATRA.

Under Th1 skewing conditions, IL-36α stimulation alone resulted in increased IFNγ expression as expected (Supplementary Fig. 6A). In contrast, and in agreement with previous reports, ATRA inhibited Th1 polarisation, resulting in a marked reduction in total IFNγ^+^ cells, alongside an increased population of IFNγ^-^α4β7^+^ cells.^32^ These data indicate that ATRA treated CD4^+^ T cells are being directed away from committing to a proinflammatory Th1 phenotype, while simultaneously gaining gut homing potential. Interestingly, when these cells were activated in the combined presence of IL-36α and ATRA, they maintained enhanced Th1 polarisation while also increasing expression of α4β7 (Supplementary Fig. 6A).

While IL-36α inhibited the generation of a FOXP3^+^ Treg population, ATRA directly enhanced iTreg differentiation, in addition to inducing expression of α4β7. Intriguingly, IL-36α stimulation in the presence of ATRA, also resulted in a diminished total population of FOXP3^+^ Tregs but enhanced the expansion of a population of α4β7^+^ FOXP3^−^ non-Treg cells (Supplementary Fig. 6B). These data indicate that IL-36 has the capacity to redirect cells towards a pro-inflammatory FOXP3 negative gut homing T cell phenotype even in the presence of ATRA.

Collectively, these data demonstrate that IL-36 has the potential to enhance expression of α4β7on CD4^+^ T cells. In addition, IL-36α stimulation can overcome the immunosuppressive effects of RA while promoting the acquisition of a gut homing phenotype by CD4^+^ T cells.

### IL-36α promotes the generation of human colitogenic CD4^+^ T cells

As we have identified a potential mechanism whereby IL-36 promotes colitis via the enhanced gut homing potential of pro-inflammatory Th1 cells in mice, we next sought to investigate what effect IL-36 has on human CD4^+^ T cell responses.

**Fig. 6:**
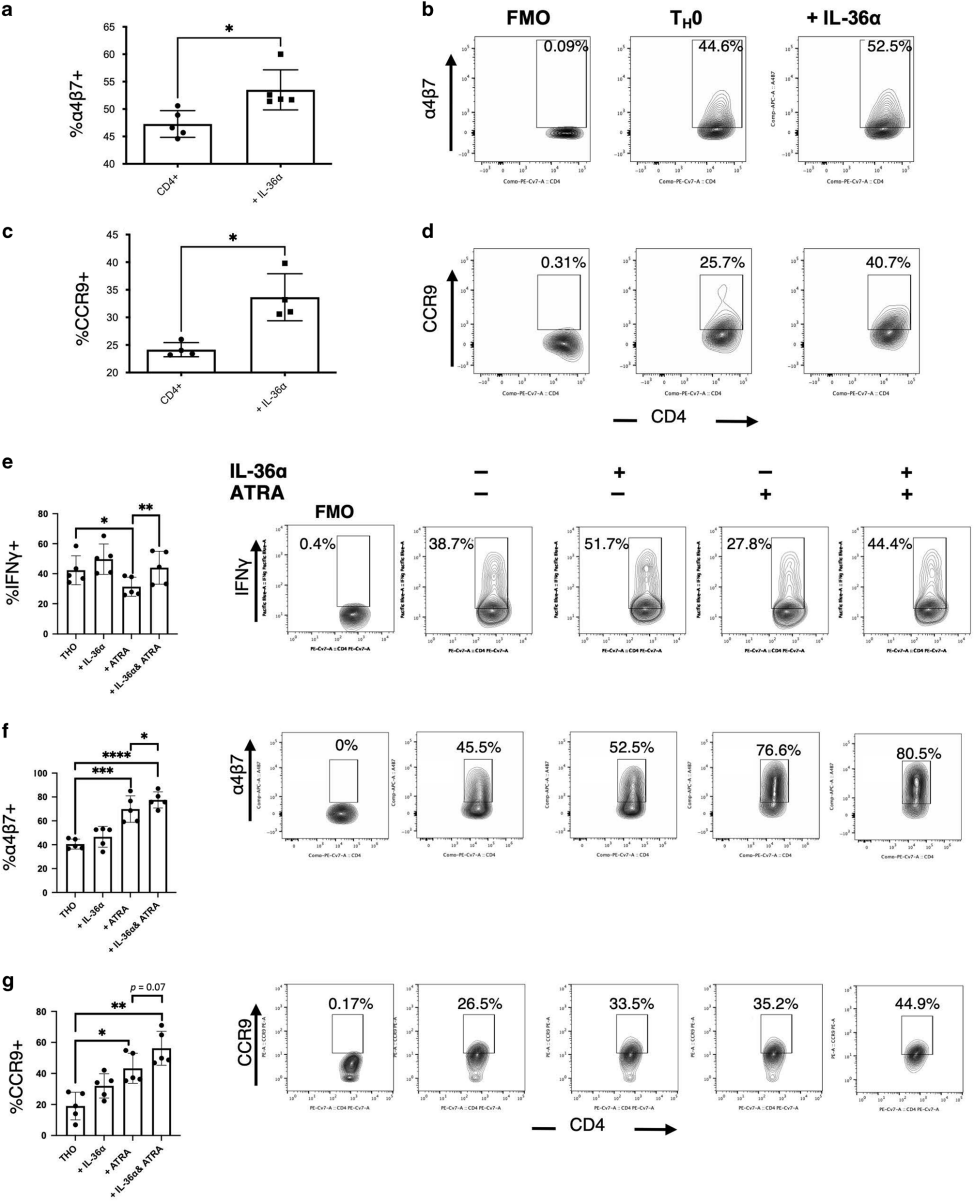
IL-36α promotes the activation of human CD4^+^ T cells with colitogenic potential. CD4^+^ T cells were magnetically isolated from human PBMCs, activated with anti-CD3 and anti-CD28 activation beads, stimulated with IL-36α (100 ng/ml) and incubated at 37 ^o^C for 120 h. Relative levels of α4β7 (**a**, **b**)(*n* = 5) and CCR9 (**c**, **d**)(*n* = 4). CD4 + T cells activated in the presence of IL-36α (100 ng/ml) +/− ATRA (10 nM) for 72 h and subsequently analysed for induced expression of IFNγ (**e**), α4β7 (**f**) and CCR9 (**g**) by flow cytometry (*n* = 5). Statistical analysis performed by Mann–Whitney U Test, *p < 0.05, **p < 0.01, ***p < 0.001, ****p < 0.0001.

To address this question CD4^+^ T cells were isolated from human PBMCs and activated with anti-CD3/anti-CD28 activation beads and stimulated in the presence or absence of IL-36α for 120 h. Activated human CD4^+^ T cells stimulated with IL-36α significantly upregulated expression of α4β7 and CCR9 (Fig. 6a–d). Furthermore, stimulation with IL-36α in the presence of ATRA, led to significantly enhanced expression of IFNγ, α4β7 and CCR9 (Fig. 6e–g). These data agree with our studies using murine cells and demonstrate that IL-36α is capable of inducing a pro-inflammatory gut homing phenotype in human CD4^+^ T cells increasing the rationale for targeting IL-36 in the clinic to inhibit T cell mediated inflammation in IBD.

## Discussion

Recently there has been significant interest in defining the role of IL-36 family cytokines in the setting of intestinal inflammation and in particular, in the context of IBD. While some studies have identified a role for these cytokines in resolving acute inflammation in preclinical models of colitis, many others have pointed towards a role in driving disease pathogenesis.^15–21^ As reported for other IL-1 family cytokines, these apparent dichotomous effects may reflect the stage of disease being investigated.^43^ Previously, we demonstrated elevated IL-36α expression in the colonic mucosa of newly diagnosed, treatment naïve, paediatric UC patients. We have now demonstrated that IL-36α protein is also elevated in patients’ serum with increased IL-36R expressing CD3 T cells in the colons of IBD patients indicating that IL-36 activity and IL-36 responsive T cells may play an important role during disease pathogenesis.

Despite advances in our understanding the role these cytokines play in the intestine, much remains to be uncovered with respect to the cellular mechanisms through which IL-36 influences intestinal inflammation. Significantly, we have also identified T cells as a potential responsive cell type in the inflamed colonic mucosa of newly diagnosed patients. Although IL-36 ligands have been reported to have the capacity to activate various haematopoietic and parenchymal cell types in the inflamed intestine, it is well established that IL-36 can potently direct the differentiation of CD4^+^ T helper cell responses in mice.^15,16,18^ Indeed, in studies using *Il36r*^−*/*−^ mice, IL-36 cytokines have been reported to enhance mucosal Th1 and Th9 responses, while inhibiting the differentiation of Treg cells, in driving intestinal inflammation in the *C. rodentium* infection and oxazolone models of colitis.^15,18^ Significantly, in this study we have identified T cells as a potential responsive cell type in the inflamed colonic mucosa of newly diagnosed patients raising the potential for roles for IL-36 activation of T cells in the genesis of intestinal inflammation in humans.

In order to specifically interrogate the role of IL-36 driven CD4^+^ T cell responses, we examined the ability of effector naïve *Il36r*^*−/−*^ CD4^+^ T cells to promote disease in the *Rag1*^*−/−*^ adoptive transfer model. Disease progression in this model is associated with the generation of colitogenic Th1/Th17 type responses. Interestingly, it has previously been reported that transferred wild type effector naïve CD4^+^ T cells can also differentiate de novo into FOXP3^+^ Treg cells in this setting,^29^ although such a response is insufficient to inhibit disease progression. In agreement with studies by Harusato et al.^18^ we report that deficiency of *Il36r* expression on transferred effector naïve CD4^+^ T cells significantly attenuated disease. Moreover, we have identified that this protection occurred in association with reduced peripheral Th1 responses and significantly enhanced differentiation of FOXP3^+^ Tregs, mirroring the instructive effects of IL-36 cytokines on CD4^+^ T cell differentiation in vitro (Fig. 3 and Supplementary Fig. 2).

While the alterations in proinflammatory T cell responses we observed could alone result in protection from disease, it was also observed that less severe colitis occurred in association with significant lymphadenopathy and splenomegaly in mice transferred with *Il36r*^*−/*−^ T cells. We therefore investigated whether loss of *Il36r* expression impacted trafficking of CD4^+^ T cells towards intestinal tissues. While no significant effects on CD4^+^ T cell expansion were observed in the periphery, there was a clear accumulation of transferred CD4^+^ T cells evident in both the spleen and mLNs with a concomitant decrease in numbers observed at the site of inflammation in colonic tissue. While multiple mechanisms have been reported to regulate T cell homing towards intestinal tissues, induced expression of the integrin α4β7 and chemokine receptor CCR9 are two of the most extensively characterised.^31^ Expression of α4β7 plays a central role in facilitating T cell infiltration along the intestine through interaction with its ligand, MADCAM-1 on the endothelial cell surfaces.^31^ In contrast, CCR9 activity appears to play a more prominent role in mediating the trafficking of T cells towards the small intestine.^31,44^ In the adoptive transfer model of colitis used in this study, α4β7 expression has been reported to be induced in transferred T cells in the spleen and mLN, before being downregulated on cells which entered the colon,^45^ while blockade of α4β7 activity has been shown to ameliorate disease in this model.^46^ Interestingly, we observed significantly reduced levels of α4β7 expression on transferred *Il36r*^*−/*−^ CD4^+^ T cells in the spleen, indicating that IL-36 cytokines can promote the expression of this integrin in vivo (Fig. 5). In agreement with previous reports, the levels of α4β7 expression were highest on CD4^+^ T cells in the mLNs,^45^ although somewhat surprisingly no differences were observed on *Il36r*^*−/*−^ CD4^+^ T cells at this site. This observation may be reflective of the later timepoint (at week 4 versus at week 1 for the spleen) which was required to detect sufficient numbers of transferred T cells in the mLNs and colon for meaningful analysis. Alternatively, these data may also indicate that other unidentified signals can induce α4β7 expression on the lower numbers of *Il36r*^*−/*−^ CD4^+^ T cells detected in gut draining lymph nodes. Interestingly, while most research has focused on the importance of α4β7 expression in the GALT, its induced expression on T cells has been reported to be dependent on the dose and duration of antigen exposure, as well as the availability of additional signals acting through the Retinoic Acid Receptor.^47^ Significantly, these effects have also been reported to take place in the spleen, although the implications of this for T cell homing to intestinal tissues remains to be determined.^45,47,48^ Our observations were mirrored in vitro, where IL-36 stimulation was also found to drive α4β7 expression on both human and murine CD4^+^ T cells, when stimulated in the presence of RA.

Retinoic acid (RA) has been extensively characterised as a key mediator in promoting the expression of α4β7 and CCR9 and the development of a gut homing phenotype in CD4^+^ T cells.^31,32^ However, it is striking that while RA acts as a protolerogenic factor in terms of its effects on T cell polarisation, it acts opposingly to IL-36 stimulation in this regard.^32^ Indeed, stimulation with IL-36 is sufficient to overcome the protolerogenic effects of RA on CD4^+^ Th cells, while further enhancing the expression of α4β7 (Supplementary fig. 6). These data may suggest that a balance between relative levels of RA and IL-36 may play an important role in defining the gut homing capacity of Tregs versus proinflammatory Th cells. Given that IL-36 levels are elevated in IBD patients, this may be of particular relevance in driving disease pathogenesis and an area for further investigation.

Our data reported here, identify a novel mechanism whereby IL-36 cytokines act to promote the development of CD4^+^ T cell mediated intestinal inflammation. The role of these cytokines in acting, uniquely among related IL-1 family members, to imprint a colitogenic phenotype on CD4^+^ T cells provides further validation towards ongoing efforts aimed at targeting this pathway in IBD and also indicates that such strategies may be particularly beneficial during the earliest phases of disease onset.

## Methods

### Study subjects

All human samples were provided with consent/assent from paediatric IBD patients and control participants recruited in the Determinants and Outcomes of Children and Adolescents with IBD study (DOCHAS) at the gastroenterology unit at Our Lady’s Children’s Hospital (Crumlin, Ireland). All patients have undergone diagnostic evaluation according to international paediatric standards (Porto criteria) following which, each case was rigorously phenotyped using the paediatric-specific Paris classification of IBD. Established paediatric evidence-based indices are used to document clinical disease activity—a Physician Global Assessment score for each case; the Paediatric Crohn’s Disease Activity Index for CD and Paediatric Ulcerative Colitis Activity Index for UC. Rectal biopsies and serum were obtained from patients as part of routine diagnostic evaluation upon initial enrollment in patients who were treatment naive. Patients, who were initially enrolled with suspected IBD, but subsequently not diagnosed with disease, comprise the control population. The analysis of IL-36 family protein expression in intestinal tissue specimens and serum samples from these participants is under approval from the institutional Research Ethics Committee (GEN/193/11), and included 96 participants (CD, *n* = 42; UC, *n* = 31; Ctrl, *n* = 23). Specific Patient details are included in Table 1.

**Table 1:** Clinical characteristics of paediatric patients included in the study.

Patient information		Numbers
Disease Groups	UC	31
	CD	42
	Ctrl	24
Sex	Female	34
	Male	63
Age	0–5 years	12
	5–10 years	12
	10–15 years	61
	15+	12
CD Phenotype	L1	1
	L2	9
	L3	18
	L4a	7
	L4b	2
	PA disease only	0
	Oral Disease Only	0
	Undefined	5
UC Phenotype	E1	3
	E2	4
	E3	0
	E4	24

CD Classification:

L1: limited terminal ileal disease.

L2: isolated colonic disease L3: ileal-colonic disease.

L4a: disease proximal to the ligament of treitz.

L4b: disease distal to the ligament of treitz PA: perianal disease.

UC Classification:

E1: proctitis only.

E2: left-sided disease.

E3 extensive disease.

E4: pancolitis.

### Mice

Wild-type (wt) C57BL/6 mice, C57BL/6-Cd45.1 mice (Jackson laboratories, No. 002014), IL-36 Receptor knockout mice (Il36r^−/−^) (Amgen), and Rag1^−/−^ mice were bred and housed under specific pathogen-free conditions in a temperature-controlled unit with a 12 h light/dark cycle at the Comparative Medicine Unit in Trinity Translational Medicine Institute, St. James Hospital (Dublin, Ireland). Water and food were provided ad libitum. All mice used were aged between 6 and 10 weeks old and experiments were performed under license from the Irish Health Products Regulatory Authority (Project No. AE19136/P036) and in compliance with Irish Department of Health regulations (license number B100/4272), with approval by the institutional ethical review boards.

### ELISA

ELISA kits for mouse IFNγ and IL-17a were purchased from eBioscience (Thermo Fischer, UK) and performed in accordance with manufacturer’s instructions using Corning^®^ High Binding ELISA plates (Merck, CLS9018). Kits for human IL-36α, IL-36β, IL-36γ and IL-36Ra were also purchased from BioLegend (London, U.K.) and mybiosource.com (California, USA) and performed in accordance with manufacturer’s instructions. All ELISAs were analysed using a Synergy MX microplate reader (BioTek).

### CD4^+^ T_H_ cell culture and differentiation

Naïve CD4^+^ T cells were purified from mice spleens and lymph nodes using positive selection by magnetic beads (CD4 L3T4 Kit, Miltenyi Biotec, UK). The purified T cells were activated by plate-bound αCD3ε (1 μg/ml; 2C11) and αCD28 (3 μg/ml; 37.51), stimulated IL-36α +/− ATRA, and cultured for 48 h under T_H_0 conditions, 72 h under T_H_1 conditions (IL-12 (20 ng/ml) + αIL-4 (11B1) (10 μg/ml), 72–96 h under T_H_17 conditions (αIFNγ (10 μg/ml) + αIL-4 (5 μg/ml) + IL-6 (20 ng/nl) + TGFβ(5 ng/ml)), and 96–120 h under iTreg conditions (TGFβ (5 ng/ml)) at 37 °C.

Human CD4 + T cells were isolated from PBMCs using negative magnetic selection (CD4 T cell isolation Kit, human, Miltenyi Biotec, UK), activated with αCD3/αCD28 activation beads per manufacturer’s instructions (Treg expansion kit, Miltenyi Biotec, UK) and incubated for 120 h at 37 °C. Cells were then stimulated with IL-36α +/− ATRA. Recombinant IL-36α was purchased from R and D (Abingdon, U.K.).

### Flow cytometry

Intracellular protein expression was evaluated by first restimulating the cells with phorbol-12-myristate 13-acetate (PMA)(10 ng/ml) (Sigma Aldrich), Ionomycin (1 ug/ml) (Sigma Aldrich) and Brefeldin A (5 ug/ml) (eBioscience) for 4–6 h at 37 °C. A FOXP3 staining buffer set (eBiosciences/Thermo Fischer, UK) was used in accordance with manufacturer’s instructions to fix and permeabelise cells to facilitate detection of intracellular cytokines and transcription factors. Initial gating was performed on live cells using Live Dead fixable Aqua Dead Cell stain kit (Invitrogen). Fc block (93) and all fluorescence dye labelled antibodies antibodies used (αCD3ε (2C11), αCD4 (GK1.5), αIFNγ (XMG1.2), αIL-17a (17B7) αFOXP3 (NRRF-30; FJK-16S), anti - α4β7 (DATK32), αCCr9 (CW-1.2), αCD25 (PC61.5), αCD45Rb (C363.16 A) and αCD45.1 (A20) were purchased from eBiosciences (Thermo Fischer, UK) and R and D. Multi-parameter analysis was performed on an LSR/Fortessa (Becton Dickinson Biosciences (BD)) and analysed using FlowJo software (Tree Star). Cell sorting was performed using a FACs Melody (BD) or MOFLO XDP (Beckman) cell sorter.

### T cell transfer mediated colitis

FACS-sorted CD4 + CD25 − CD45RB^hi^ T effector cells from *wt* mice or *Il36r*^*−/−*^ mice (5 × 10^5^) were injected *i.p*. into *Rag1*^*−/−*^ recipient mice. Clinical disease progression was measured by % weight loss compared to original weight. Cut off point of 20% loss of original weight. Colons, spleens and mesenteric lymph nodes were harvested from recipient *Rag1*^−*/*−^ mice 4 weeks’ post-transfer, when clinical signs of colitis were evident. For histology, tissue was fixed overnight in 10% formalin before dehydration and embedding into paraffin blocks. Sections were subsequently cut and stained with haematoxylin and eosin. Histological scoring was performed in a blinded fashion using a system described previously.^49^ For co-adoptive transfer experiments, equal numbers (2.5 × 10^5^) of *wt CD45.1* and *Il36r*^*−/*−^ cells were injected i.p. into *Rag1*^−/−^ recipients and analysis of transferred T cells was undertaken after 2 weeks. To mitigate potential confounding effects of microbiota, for each transfer experiment, littermate *Rag1*^*−/*−^ mice were used as recipients for either *wt, Il36r*^*−/*−^ or mixed populations of T cells. Recipient mice were co-housed together irrespective of source of donor cells post transfer.

### Isolation of colonic lamina propria leukocytes

Colons were excised, opened longitudinally, and washed of fecal contents with PBS. They were then cut in pieces (0.5–1 cm), transferred to a 50 ml conical tube containing HBSS (Sigma Aldrich) supplemented with 5% FBS (Gibco) and 5 mM EDTA (e8008, Sigma Aldrich), and shaken horizontally at 37 °C for 20 min at 200 rpm. The tissue was then washed with HBSS to remove residual EDTA, cut into 1 mm pieces, and transferred to a 50 ml conical tube containing HBSS supplemented with 5% FBS, 1.6 mg/ml Collagenase D (11088858001, Roche, Sigma Aldrich), and 40 μg/ml DNase I (D4263, Sigma Aldrich). Tube was then horizontally shaken at 37 °C for 45 min at 200 rpm. After digestion, the tube was vortexed for 10 s to ensure thorough dissociation of remaining intestinal tissue, all content passed through a 100 µm cell strainer and cell suspensions pelleted by centrifugation at 2500 *rpm*. Live cells were then counted by Trypan Blue exclusion (Sigma Aldrich).

### Histopathology

Approximately 5 mm sections of distal colon and small intestine were harvested, fixed in 10% phosphate buffered formalin and embedded in paraffin. 4 μM sections were cut, stained with hematoxylin and eosin (H and E).

Images were captured using an Inverted Phase Contrast Fluorescent Microscope (Leica DMLB). Captured images were scored blindly, graded semi-quantitatively from 0 to 5 based on the protocol established by Asseman et al.^50^

### Immunofluorescence

Paraffin embedded blocks of colon biopsies from children affected with CD (*n* = 4), UC (*n* = 6) or healthy controls (*n* = 5) were obtained from Our Lady’s Children’s Hospital Crumlin. Blocks were sectioned in a microtome and 5 μm thickness sections were mounted in Superfrost Plus adhesion slides (Thermo Scientific, Braunschweig, Germany). Antigen retrieval was performed using 10 mM citrate buffer in a microwave. Tissue was probed with anti-human CD3 FITC (UCHT1)(eBioscience, Thermofisher, 11-0038-80), Anti-IL1RL2 (IL-36R)(ThermoFisher, UK) or Rabbit Anti-IgG isotype control (Thermofisher, UK) at dilutions of 1:1000 and 1:500 respectively. The secondary antibody used was Goat anti-Rabbit IgG (H + L) Highly Cross-Adsorbed Alexa Fluor 594 (ThermoFisher, UK) and the mounting media SlowFade Gold antifade Mountant with DAPI (ThermoFisher, UK). Specificity of Anti-IL-36R staining was confirmed using an isotype control (Supplementary Fig. 7). Images were taken using a confocal microscope Zeiss LSM700. Positive cells stained with anti-human CD3 or/and anti-IL1RL2 were quantified using ImageJ tool Fiji. An average of three different stacks per sample were analysed statistically.

### Analysis of single cell RNA sequencing data

Single cell RNA sequencing (scRNAseq) data from the colonic mucosa of healthy donors and patients with UC,^26^ was downloaded from the Single Cell Portal (SCP; singlecell.broadinstitute.org). Using SCP’s meta data and annotation, IL36R expression in individual T cell subsets defined by Smillie, Biton and Ordovas-Montanes et al.^26^ from healthy, non-inflamed, and inflamed tissues was analysed. The Seurat package (version 4.0) was used for normalisation and log-transformation of the gene expression data, and the ggplot2 package in R was used for plotting data.

### Statistical analysis

Sample size for patient’s serum analysis and mouse studies were chosen based on published studies.^15^ Post-hoc analysis of patient cohort revealed this sample size to have a power of 88.7%, with the probability of a type 1 error set at *a* = 0.05. Analysis of cytokines in patient’s serum and colonic tissue was conducted blindly, and subsequently segregated based on clinical parameters. Analysis from in vivo studies, i.e. histology, was also carried out in a blinded fashion. For all data sets, statistical analysis was performed by first analysing the data for normal distribution (Shapiro–Wilk or Kolmogorov–Smirnov test) and equality of variance (*F* test), subsequently the data was analysed by two-tailed student’s *t* test for parametric data or Mann–Whitney U test and Kruskal–Wallis *H* test for nonparametric data, as appropriate. All analysis and graph representation were performed using GraphPad Prism 6 software. Data are shown as means ± SEM and statistical details for each figure can be found in the figure legends.

## Supplementary information

Supplementary Materials
