# Supplementary material for: IL-36 cytokines imprint a colitogenic phenotype on CD4+ T helper cells
Source: Mucosal Immunol. 2022 Feb 17;15(3):491–503. doi: 10.1038/s41385-022-00488-w (PMC9038530; doi:10.1038/s41385-022-00488-w)
Supplement: Supplementary file 1 — Supplementary Materials [file 41385_2022_488_MOESM1_ESM.pdf]

Supplemental Figure 1 .

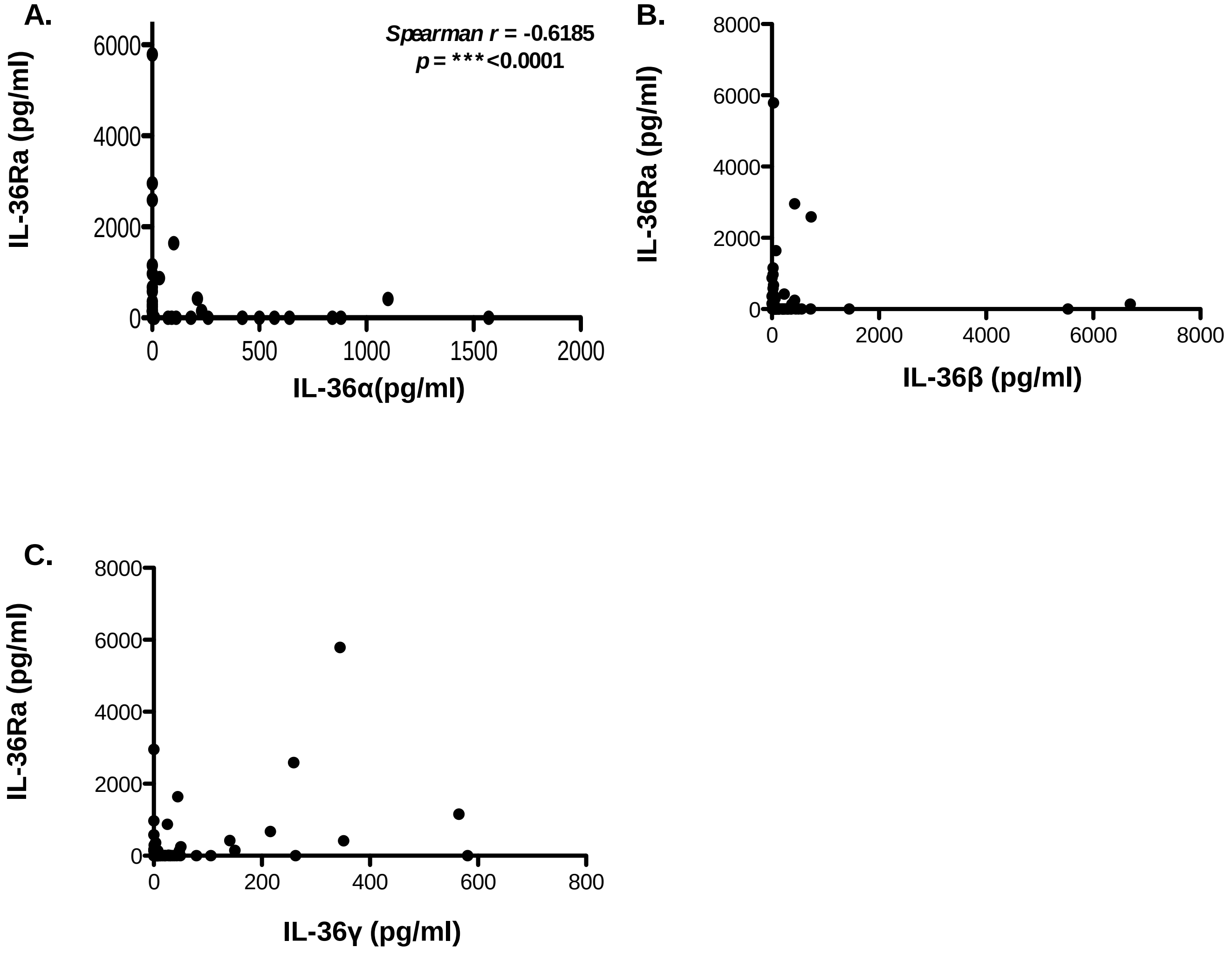

Supplemental Figure 1. IL-36α protein expression negatively correlates with IL-36Ra expression in paediatric IBD patient's serum. Spearman correlation analysis of serum from IBD patients with detectable expression of agonists, IL-36α (A), n=36; IL-36β (B), n = 89; IL-36γ (C), n=62, relative to IL-36Ra. ***p<0.001.

Supplemental Figure 2 .

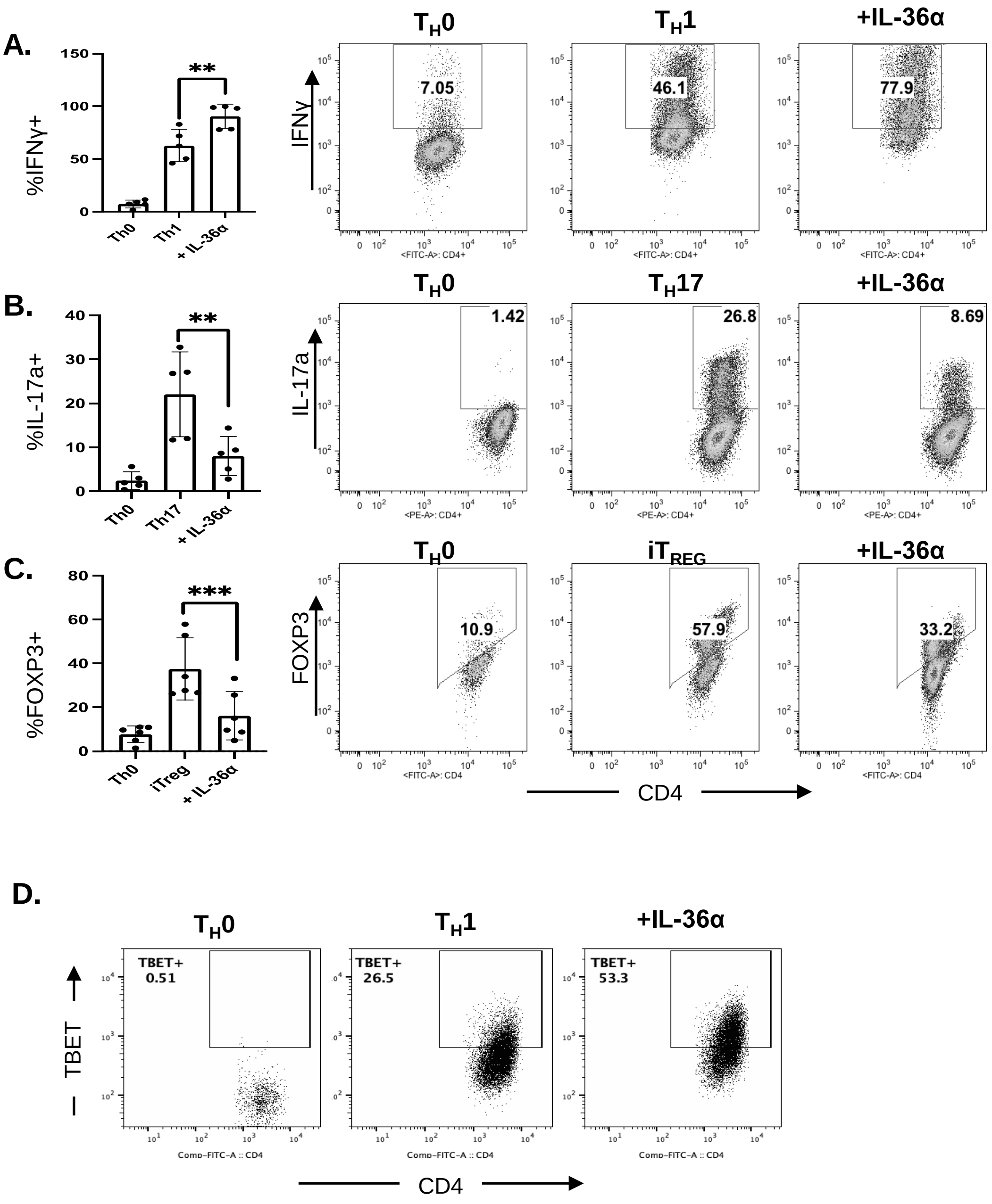

Supplemental Figure 2. IL-36α regulates CD4⁺ T cell polarisation in vitro. CD4⁺ T cells were magnetically purified from the spleens of *wt* mice and activated under the specified differentiation conditions in the presence or absence of IL-36α (200ng/ml). Cells were incubated at 37°C and analysed for intracellular expression of IFNγ (A)(n=5) & IL-17a (B)(n=5) at 72hrs, FOXP3 (C)(n=6) at 96hrs, and Tbet (D) at 24hrs by flow cytometry. Statistical analysis performed by Students T Test, **p<0.01, ***p<0.0001.

Supplemental Figure 3 .

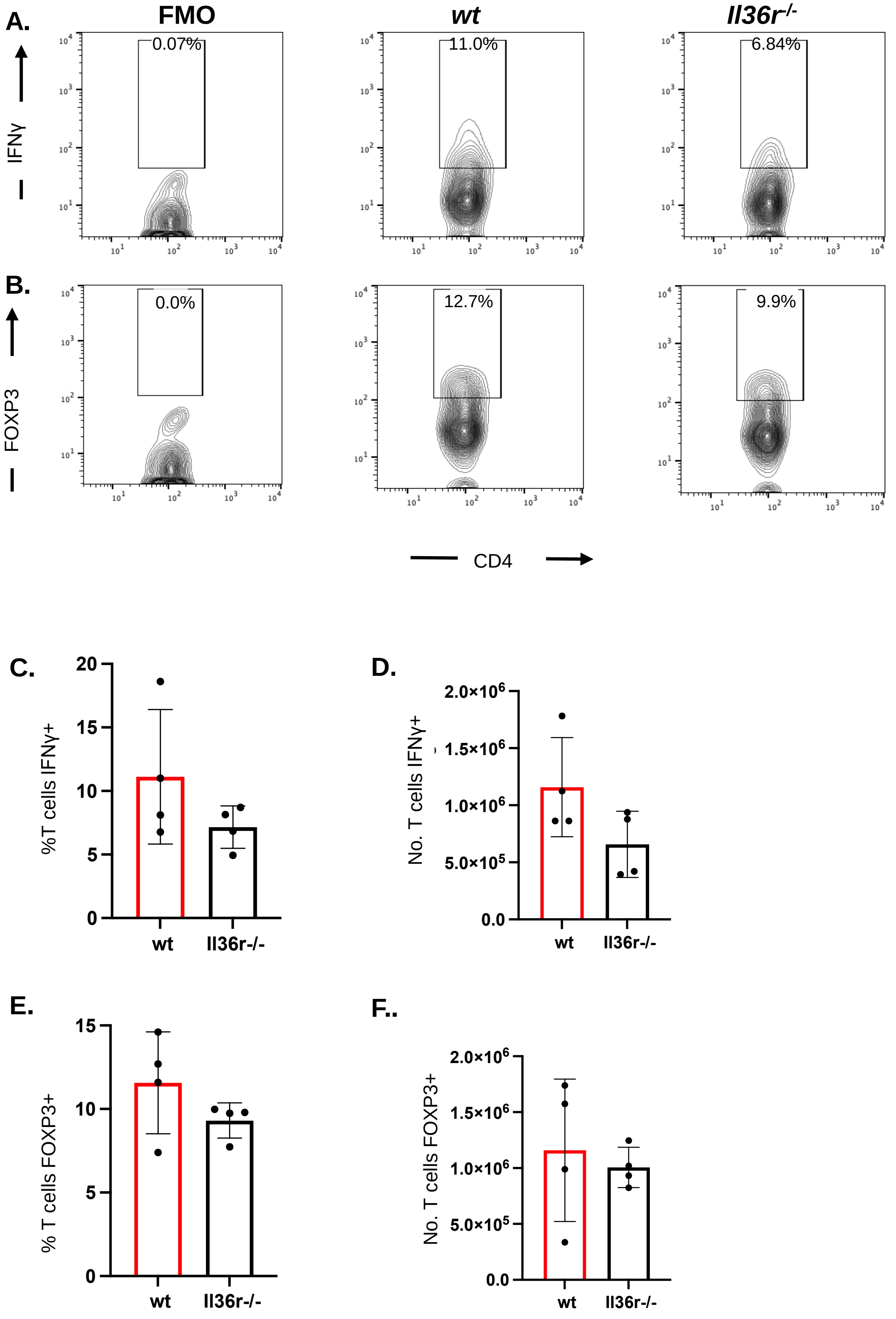

Supplemental Figure 3. *Il36r* expression does not alter Th1 and Treg population composition under homeostatic conditions. Spleens were harvested from *wt* and *Il36r-/-* mice and expression of IFNg and FOXP3 by $CD4^{+}$ T cells was analysed by flow cytometry, n = *wt*: 4; *Il36r*$^{-/-}$: 4. Statistical analysis performed by Mann Whitney U Test, *p<0.05.

Supplemental Figure 4 .

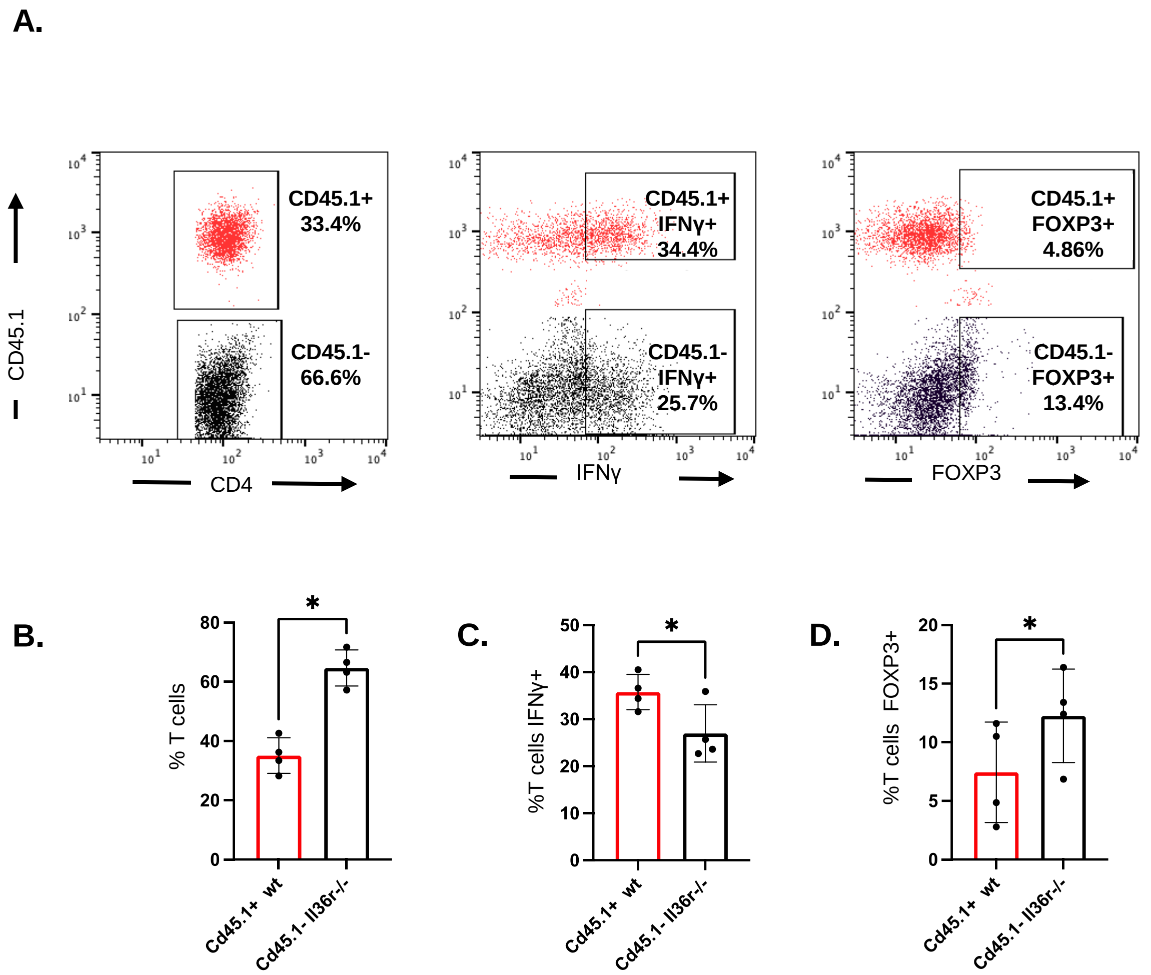

Supplemental Figure 4. Absence of *Il36r* expression on CD4⁺ T cells alters Th1 and Treg development *in vivo* in the presence of wt CD4⁺ T cells. $CD4^+CD25^-CD45Rb^{hi}$ effector T cells from donor *wt* $CD45.1^+$ mice and *Il36r-/-* $CD45.1^-$ mice were FACs purified. These cells were mixed in a 1:1 ratio and both populations were transferred by i.p. injection to the same Rag1-/- recipient mice. At week 2 the spleens were harvested for analysis of $CD4^+$ T cell CD45.1 (A&B), IFNγ (A&C), and Foxp3 (A&D) protein expression by multi-parameter flow cytometry (n = $CD45.1^+$ *wt*: 4; $CD45.1^-$ *$Il36r^{-/-}$*: 4). Statistical analysis performed by Students T Test, *p<0.05.

Supplemental Figure 5 .

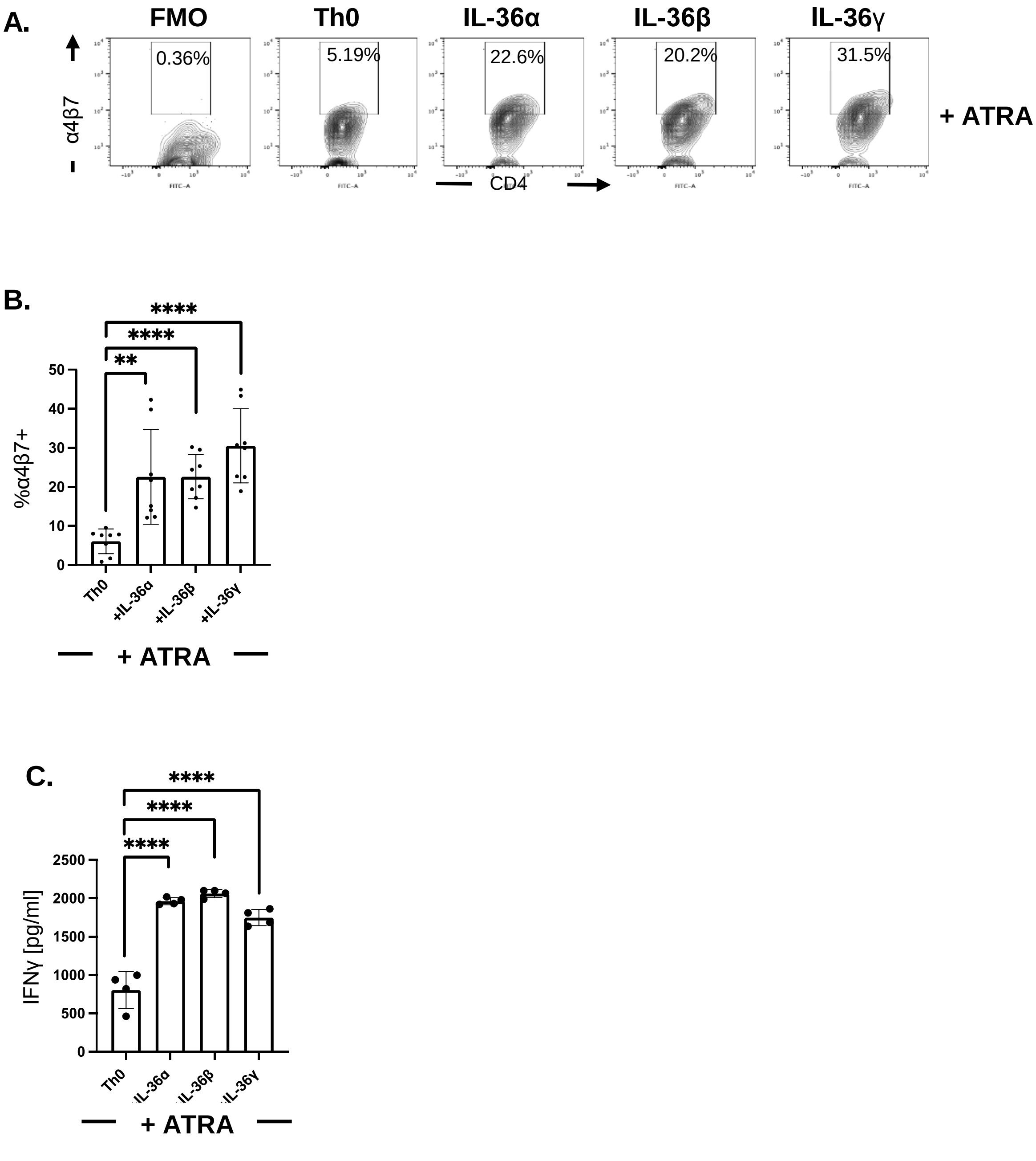

Supplemental Figure 5. IL-36 cytokine family agonists induce similar levels of α4β7 and IFNγ expression on CD4⁺ T cells in the presence of ATRA. Murine splenic CD4⁺ T cells were magnetically purified, activated with plate bound anti-CD3 & anti-CD28, and stimulated with the indicated IL-36 family cytokines; IL-36α (200ng/ml), IL-36β (200ng/ml), & IL-36γ (200ng/ml), +/- ATRA (10nM). Cells were incubated at 37°C for 72hrs and analysed for expression of α4β7 (A&B)(n=8) by flow cytometry and IFNγ (C)(n=4) by elisa. Data shown representative of 2 independent experiments. Statistical analysis performed by Students T Test, **p<0.01, ****p<0.0001.

Supplemental Figure 6.

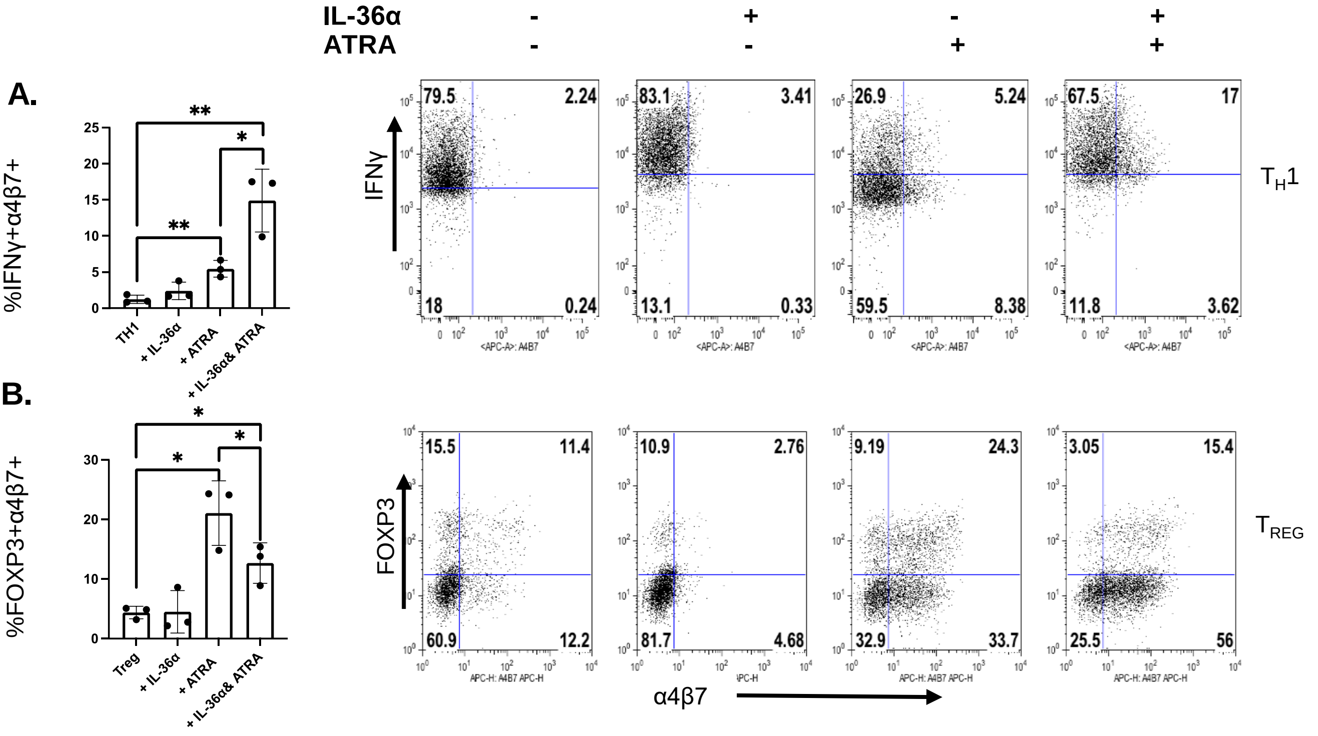

Supplemental Figure 6. IL-36α promotes the differentiation of proinflammatory CD4⁺ T cells with gut homing potential in the presence of ATRA. CD4⁺ T cells were magnetically purified from the spleens of *wt* mice and activated under either Th1 (A&C) or iTreg (B&D) differentiation conditions. Cells were either unstimulated or cultured in the presence of IL-36α (100ng/ml), ATRA (10nM) or IL-36α + ATRA as indicated . After 72-96hr, levels of coexpression of α4β7 with IFNγ (A)(n=3) and FoxP3 (B)(n=3) were determined by flow cytometry. Data is representative of 3 indpendent experiments. Statistical analysis performed by Students paired-T Test, *p<0.05, **p<0.01.

Supplemental Figure 7.

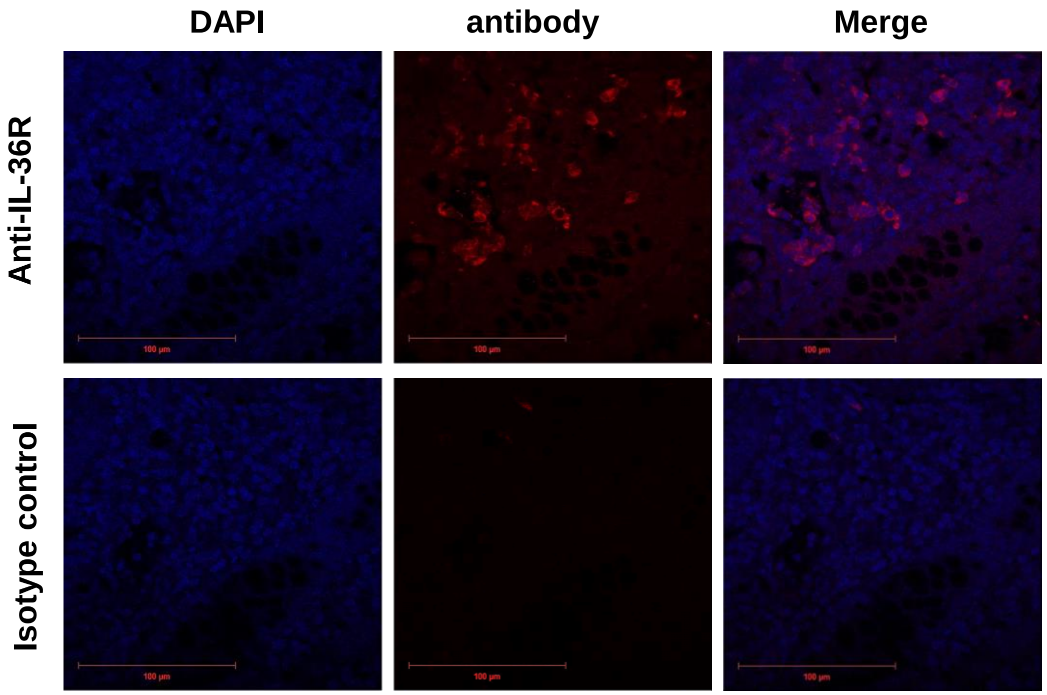

Supplemental Figure 7. Anti-IL-36R antibody specificity. Representative immunofluorescent staining of neighbouring tissue sections from a CD patient colon biopsy stained with anti-IL-36R or matching anti-IgG isotype control antibody.
